# Complicated Pregnancy: Unveiling the Dual Challenge of Acute Cholecystitis and Choledocholithiasis

**DOI:** 10.7759/cureus.55533

**Published:** 2024-03-04

**Authors:** Ketki S Dantkale, Manjusha Agrawal

**Affiliations:** 1 Obstetrics and Gynecology, Jawaharlal Nehru Medical College, Datta Meghe Institute of Higher Education & Research, Wardha, IND

**Keywords:** intervention, multidisciplinary care, management, choledocholithiasis, acute cholecystitis, pregnancy

## Abstract

Gallstone-related complications during pregnancy, though relatively rare, present significant challenges necessitating careful clinical management. Among these complications, the simultaneous occurrence of acute cholecystitis and choledocholithiasis poses a unique dual challenge, especially considering the physiological changes and fetal considerations associated with pregnancy. This case report presents the management of a 27-year-old pregnant woman with acute cholecystitis and choledocholithiasis at 32 weeks of gestation. Diagnostic imaging, including magnetic resonance cholangiopancreatography (MRCP) and endoscopic retrograde cholangiopancreatography (ERCP), played crucial roles in the comprehensive evaluation and treatment of the patient. Conservative measures followed by interventional procedures successfully addressed gallstone-related complications while considering maternal and fetal well-being. Collaborative multidisciplinary care involving obstetricians, gastroenterologists, and other specialists was essential in navigating the case's complexity. The successful outcome highlights the importance of individualized management and multidisciplinary collaboration in optimizing maternal and fetal outcomes in pregnant patients with complex gallstone diseases. This case underscores the necessity for continued research and shared clinical experiences to refine the approach to such intricate medical scenarios, ultimately enhancing the quality of care provided to pregnant individuals facing gallstone-related complications.

## Introduction

Gallstone-related complications during pregnancy, although relatively infrequent, present a unique set of challenges requiring meticulous clinical management. The physiological changes associated with pregnancy, including hormonal fluctuations and increased cholesterol saturation in the bile, contribute to the increased risk of gallstone formation and related complications [1]. Among these complications, acute cholecystitis and choledocholithiasis are complex scenarios demanding timely and tailored interventions to safeguard maternal and fetal well-being. Several studies have underscored the association between pregnancy and an elevated risk of gallstone formation, attributing it to hormonal influences, decreased gallbladder motility, and increased cholesterol secretion into the bile [1,2]. The prevalence of gallstone disease during pregnancy varies, with estimates ranging from 5% to 22%, emphasizing the need for heightened clinical awareness and vigilant monitoring in pregnant individuals [2,3].

The potential sequelae of gallstone complications during pregnancy extend beyond the immediate clinical concerns, with implications for both maternal and fetal outcomes [4]. Intrahepatic cholestasis of pregnancy, for instance, has been linked to adverse fetal complications, necessitating a comprehensive understanding of the intricate relationships between maternal physiology and fetal health [5]. Additionally, the rupture of the biliary tract during pregnancy, though rare, underscores the importance of recognizing and managing gallstone-related complications promptly [6]. To date, literature discussing the simultaneous occurrence of acute cholecystitis and choledocholithiasis during pregnancy remains limited, necessitating exploration of the diagnostic and therapeutic considerations specific to this dual challenge. This case report aims to contribute to the existing knowledge base by elucidating the complexities of managing gallstone disease in pregnant women and highlighting the multidisciplinary strategies employed for optimal patient outcomes.

## Case presentation

A 27-year-old woman sought care at the outpatient department of a tertiary care hospital, reporting a sudden onset of abdominal pain. She had a known case of acute cholecystitis with choledocholithiasis. During the history collection, it was revealed that she had been admitted to Lifecare Hospital in Nanded with complaints of amenorrhea for the past seven months. This was her second pregnancy at 32 weeks of gestational age, and she had received two doses of a tetanus toxoid injection. A physical examination revealed swelling in the lower limbs.

An ultrasound of the abdomen and pelvis was recommended upon consultation with gynecologists. The results showed oligohydramnios, a distended gall bladder with sludge and multiple calculi measuring approximately 2 x 0.3 cm, a liver abscess, mild bilateral pleural effusion, and bilateral kidney physiological hydronephrosis. Consequently, the doctor diagnosed her with gallstone disease, cholecystitis, and choledocholithiasis. The patient was advised to be admitted for further evaluation and management and was referred to a tertiary care hospital in Wardha.

**Figure 1:**
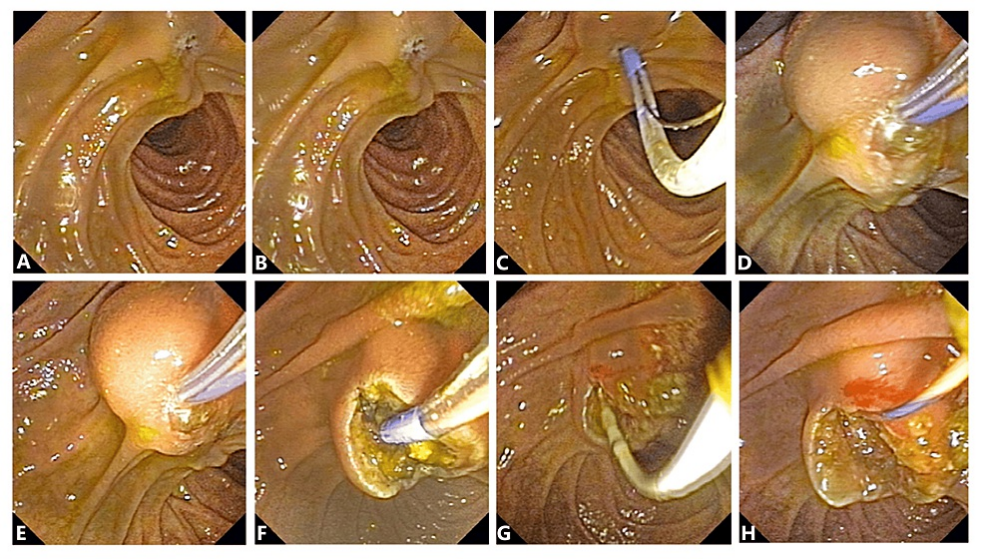
The MRCP revealed diffuse thickening of the gall bladder wall (3-4 mm), multiple calculi with focal contained perforation in the anterior wall, a dilated common bile duct (12 mm) with a calculus (13 x 8 mm) in the distal part, and cholangitic abscesses in the intra-hepatic and peri-cholecystic spaces.

Upon admission, the patient received antibiotic injections (piptaz and metronidazole) and analgesics for supportive management. The doctor recommended magnetic resonance cholangiopancreatography (MRCP) and endoscopic retrograde cholangiopancreatography (ERCP). The MRCP revealed diffuse thickening of the gall bladder wall (3-4 mm), multiple calculi with focal contained perforation in the anterior wall, a dilated common bile duct (12 mm) with a calculus (13 x 8 mm) in the distal part, and cholangitic abscesses in the intra-hepatic and peri-cholecystic spaces. Additionally, calculi were in the proximal part of the main pancreatic duct (7 mm dilation) (Figure 1). The pelvicalyceal system of both kidneys showed dilation, more pronounced on the right side, indicating hydronephrosis related to pregnancy.

An ERCP was performed under general anesthesia, revealing choledocholithiasis with cholangitis. The bile aspirate was sent for culture, and the greenish-turbid nature of the aspirate was noted. Endoscopic papillary transpapillary (EPT) procedures were performed, and stones were removed by placing a 10 Fr x 10 cm straight plastic stent in the common bile duct (CBD). Post-ERCP, the patient was observed, kept on antibiotics, and remained nil per oral for six hours. The cytopathology of the cholangitic abscess aspirate showed scattered polymorphs in amorphous proteinaceous backgrounds.

The patient and her husband were counseled about the nature of the illness and the need for further evaluation and management. The physician recommended cholecystectomy followed by stenting after seven days and discharged the patient with oral antibiotics and antacids.

## Discussion

The presented case underscores the intricate management challenges associated with acute cholecystitis and choledocholithiasis during pregnancy, emphasizing the necessity of a comprehensive and multidisciplinary approach. In pregnant women, gallstone diseases, though relatively uncommon, can lead to serious complications necessitating prompt evaluation and intervention [5]. The concurrent occurrence of acute cholecystitis and choledocholithiasis poses a dual challenge, as highlighted in this case, where the patient's advanced gestational age further compounded the complexity. The diagnostic approach in this case involved ultrasound imaging, magnetic resonance cholangiopancreatography (MRCP), and endoscopic retrograde cholangiopancreatography (ERCP). MRCP provided detailed insights into gallbladder wall thickening, multiple calculi, and cholangitic abscesses, aiding in the comprehensive evaluation of gallbladder and bile duct pathology [7]. ERCP emerged as a pivotal interventional tool, confirming choledocholithiasis and cholangitis and facilitating stone removal through endoscopic papillary transpapillary (EPT) procedures. This aligns with existing literature supporting the efficacy of ERCP in pregnant patients when conservative measures are insufficient [8].

The decision-making process for managing pregnant patients with gallstone diseases requires a delicate balance between maternal and fetal considerations. Conservative measures, including antibiotic therapy and analgesics, were initially employed in this case to ensure supportive management while minimizing potential risks to the fetus [3]. The subsequent ERCP procedure was performed carefully, considering the risks and benefits and highlighting the importance of individualized decision-making in complex pregnancies. The presence of hydronephrosis related to pregnancy further complicated the scenario, underscoring the importance of a holistic approach to managing pregnant patients with multiple comorbidities. Studies have emphasized the need for collaborative care involving obstetricians, gastroenterologists, and other specialists to optimize outcomes in complex cases [9]. The proposed treatment plan involving cholecystectomy and stenting after a seven-day interval aligns with existing literature suggesting that delaying definitive surgical intervention until after the second trimester is associated with lower risks to the fetus [10]. Counseling the patient and her husband about the nature of the illness, the proposed interventions, and the need for further evaluation and management ensures informed decision-making and active involvement in the patient's care.

## Conclusions

In conclusion, the successful management of acute cholecystitis and choledocholithiasis during pregnancy hinges on a thorough understanding of the unique challenges posed by this dual pathology. This case serves as a valuable contribution to the existing medical literature, emphasizing the significance of individualized care, multidisciplinary collaboration, and the delicate balance between maternal and fetal considerations in optimizing outcomes for the pregnant patient and her unborn child. As we navigate the complexities of such cases, continued research and shared clinical experiences will further refine our approach, ultimately enhancing the quality of care provided to pregnant individuals facing intricate medical challenges.
